# HPV caught in the tetraspanin web?

**DOI:** 10.1007/s00430-020-00683-1

**Published:** 2020-06-13

**Authors:** Jérôme Finke, Lisa Hitschler, Klaus Boller, Luise Florin, Thorsten Lang

**Affiliations:** 1 Department of Membrane Biochemistry, Life & Medical Sciences (LIMES) Institute, University of Bonn, Carl-Troll-Straße 31, 53115 Bonn, Germany; 2 Paul Ehrlich Institute, Paul-Ehrlich-Straße 51-59, 63225 Langen, Germany; 3 Institute for Virology and Research Center for Immunotherapy (FZI), University Medical Centre of the Johannes Gutenberg University Mainz, Obere Zahlbacher Straße 67, 55131 Mainz, Germany

**Keywords:** CD151, CD63, Actin, OBSL1, Papillomavirus, Pathogen endocytosis, Microdomains, Protein nanoclustering

## Abstract

Tetraspanins are master organizers of the cell membrane. Recent evidence suggests that tetraspanins themselves may become crowded by virus particles and that these crowds/aggregates co-internalize with the viral particles. Using microscopy, we studied human papillomavirus (HPV) type 16-dependent aggregates on the cell surface of tetraspanin overexpressing keratinocytes. We find that aggregates are (1) rich in at least two different tetraspanins, (2) three-dimensional architectures extending up to several micrometers into the cell, and (3) decorated intracellularly by filamentous actin. Moreover, in cells not overexpressing tetraspanins, we note that obscurin-like protein 1 (OBSL1), which is thought to be a cytoskeletal adaptor, associates with filamentous actin. We speculate that HPV contact with the cell membrane could trigger the formation of a large tetraspanin web. This web may couple the virus contact site to the intracellular endocytic actin machinery, possibly involving the cytoskeletal adaptor protein OBSL1. Functionally, such a tetraspanin web could serve as a virus entry platform, which is co-internalized with the virus particle.

**Electronic supplementary material:**

The online version of this article (10.1007/s00430-020-00683-1) contains supplementary material, which is available to authorized users.

## Introduction

Tetraspanins are small membrane proteins interacting among each other and other types of membrane proteins, e.g., receptors, adhesion molecules, or members of the immunoglobulin superfamily. By these interactions, they form so-called tetraspanin enriched microdomains (TEMs) [1]. Because most tetraspanins localize to the cell surface, they are also referred to as master organizers of the plasma membrane. As a result, they play roles in many cellular functions occurring at the cell membrane, such as adhesion, endocytosis, signaling, and pathological processes like pathogen entry [2].

The involvement in pathogen entry is best studied for viral infection. Several reports document that TEMs modulate the cell entry for different types of viruses, including the human papillomavirus (HPV) [3], coronavirus [4], influenza A virus [5], hepatitis C virus [6, 7] and human immunodeficiency virus [8, 9]. In the case of HPV infection, TEMs organize viral entry platforms with components for virus binding and endocytosis [10]. However, tetraspanins may be as well binding receptors, as in the case of CD81 that directly binds to hepatitis C virus. Direct interactions may be very specific, as human CD81 variants are related with different susceptibilies for infection with different HCV genotypes [11].

High-risk human HPVs, like HPV16, are involved in a variety of cancers, notably cervical and anogenital cancers [12]. In the beginning, the study of HPV cell entry was limited by the availability of viral particles. This changed by developing an efficient system involving intracellular assembly of pseudovirion particles (PsVs) with an encapsidated reporter plasmid [13].

The involvement of tetraspanin CD151 (TSPAN24) crowds that form during the entry of HPV is well-studied. Employing total internal reflection fluorescence (TIRF)-microscopy, a method that enables imaging plasma membrane events without fluorescence background from the cell interior, it was previously shown that CD151 forms large aggregates at the basal cell membrane at sites where HPV16 PsVs are present [3]. Disappearance of the viral particle from the evanescent-field, due to movement towards the cell interior, is accompanied by the disappearance of the CD151 aggregate. This suggests that the large aggregates are co-internalized with the viral particles [3]. Another study employing superresolution microscopy shows that CD151 nanoclusters associated with PsVs become brighter over time and that PsVs are preferentially co-internalized with large CD151 clusters [14].

Apart from CD151, which is essential for HPV infection, HPV16 PsVs also associate with other tetraspanins, such as CD63 (TSPAN30) [15, 16] and CD81 (TSPAN28) [17]. CD63 is required for intracellular trafficking of the virus and, therefore, is also a key player in the HPV infection process [15, 18, 19], whereas CD81 plays a less prominent role [15, 19]. Tetraspanin CD9 (TSPAN29), which associates with proviral factors such as ADAM proteases [20, 21], seems to play an indirect role in HPV16 entry [21].

Altogether, these observations suggest that a special type of TEM is forming for viral entry [22]. Virus-platform association and co-internalization indicate a role of the platform in linking the extracellular viral contact site to the intracellular endocytic machinery. For the entry of HPV16 it has been suggested that an endocytic pathway is used, which is ligand-induced and related to macropinocytosis, dependent on actin but not on clathrin [15, 23–25]. Some kind of molecule must connect this endocytic machinery to intracellular dynamics. OBSL1 may be such a candidate. It is thought to be a cytoskeletal adaptor protein [26], is required for HPV16 endocytosis, and colocalizes with CD151 [27].

To shed some light on these questions, we studied by microscopy the spatial relationship between CD151 and CD63, two tetraspanins known to be required for HPV infection of HaCaT keratinocytes, one of the most widely used keratinocyte models for studying HPV infection [28]. Moreover, we wondered to which extent actin is associated with the entry platforms and whether obscurin-like protein 1 (OBSL1) is also present, which based on its role as a cytoskeletal adaptor protein, could link the endocytic machinery to the virus contact site.

## Materials and methods

### Antibodies and plasmids

As primary antibodies we used the HPV16-L1 detecting antibody 16L1-312F (mouse monoclonal, diluted 1:200; [29]) and a rabbit polyclonal antibody raised against the V5-tag (diluted 1:5000; cat# ab9116, Abcam, Cambridge, UK). In addition, we employed GFP-Booster Atto488 (Chromotek, cat# gba488, Planegg-Martinsried, Germany) and RFP-Booster Atto594 (cat# rba594, Chromotek, Planegg-Martinsried, Germany), both diluted 1:200. As secondary antibodies we used AlexaFluor488 labelled donkey anti-mouse (cat# A-21202, Carlsbad, CA) and AlexaFluor594 labelled donkey anti-rabbit (cat# ab150064, Abcam). For transfection, we used the previously described plasmids encoding CD63-GFP [18], V5-OBSL1 [27, 30] and CD151-RFP [31].

### Cell culture and transfection

HaCaT and HepG2 cells were obtained from Cell Lines Services (Eppelheim, Germany). HaCaT cells were grown in high glucose (4.5 g/l) DMEM (cat# P04-03550 PAN Biotech, Aidenbach, Germany) supplemented with 10% fetal bovine serum (cat# S0615, Biochrom AG, Berlin, Germany) and 1% penicillin–streptomycin (cat# P06-07100, PAN Biotech). HepG2 cells were grown in MEM (cat# P04-08509, PAN Biotech) supplemented with 10% fetal bovine serum and 1% penicillin–streptomycin. Both cell lines were kept at 37 °C and 5% CO_2_.

For transfection, cells were trypsinated with trypsin solution (cat# P10-0231SP, PAN Biotech) for about 2 min (HepG2 cells) or 10 min (HaCaT cells) at 37 °C. Trypsin activity was stopped by adding growth medium. HaCaT cells were washed in DPBS (cat# P04-36500, PAN-Biotech) and resuspended in cytomix solution (120 mM KCl, 10 mM KH_2_PO_4_, 0.15 mM CaCl_2_, 2 mM EGTA, 5 mM MgCl_2_, 25 mM HEPES–KOH, pH 7.6). For one transfection, 2 × 10^6^ cells were mixed with 15 µg plasmid DNA or, in case of double transfection, with 15 µg plasmid DNA of each construct. Afterwards, cells were electroporated using the Gene pulser Xcell electroporation system (Bio-Rad, Hercules, CA) employing the following settings: 200 V, 950 μF and 200 Ω.

HepG2 cells were transfected using the Neon electroporation system (Thermo Fisher, Waltham, MA) essentially as described previously [32].

Cells were seeded onto glass-coverslips (~ 3 × 10^5^ cells/coverslip) coated with 100 µg/ml poly-L-lysine (cat# P6282, Sigma-Aldrich, St. Louis, MO) placed in 6-well plates and incubated for 24 h in growth medium at 37 °C until using them for experiments.

### Sample preparation

Cells were incubated with 4 × 10^7^ viral genome equivalents (vge) (PsVs were prepared as previously described [10]) per well in culture medium without antibiotics for 3 h at 37 °C. Cells were washed twice in DPBS and either fixed directly or after membrane sheet generation. Membrane sheets were essentially generated as previously described [33] in sonication buffer (120 mM potassium glutamate, 20 mM potassium acetate, 10 mM EGTA, 20 mM HEPES, pH 7.2) applying a 100 ms ultrasound pulse at the center of the coverslip. The samples were fixed at room temperature in 4% PFA in PBS (phosphate buffered saline) for 30 min. Fixative solution was removed and remaining PFA was quenched with 50 mM NH_4_Cl in PBS for 30 min. Samples were permeabilized by incubation with 0.2% Triton X-100 in PBS for 1 min (membrane sheets) or 2 min (cells). Then, samples were blocked in 3% BSA in PBS for 30 min. Incubation with primary antibody was performed in 3% BSA in PBS for 1 h at room temperature and samples were washed three times with PBS. The samples were incubated for 1 h at room temperature with secondary antibodies diluted 1:200 with 3% BSA in PBS. In experiments using GFP-Booster, the GFP-Booster was added to the secondary antibody solution. For Fig. 3, RFP-Booster was added as well and the primary antibody incubation step was omitted. Phalloidin-iFluor647 (cat# ab176759, Abcam) was used for F-actin staining. A 100 × stock solution was prepared in DMSO according to the manufacturer’s instructions and added as a 1:100 dilution during the secondary antibody incubation. Afterwards, samples were washed three times with PBS and mounted on microscopy slides with ProLong^®^ Gold antifade mounting medium (cat# P36930, Invitrogen), cured overnight, and sealed with nail varnish.

### Confocal and STED microscopy

A 4-channel easy3D super-resolution STED optics module (Abberior Instruments, Göttingen, Germany) coupled with an Olympus IX83 confocal microscope (Olympus, Tokyo, Japan) and equipped with an UPlanSApo 100 × (1.4 NA) objective (Olympus) was used for confocal and STED microscopy (available in the LIMES imaging facility). Atto488 and Alexa488 were excited with a 485 nm laser and recorded with combined 500–520 nm and 532–558 nm filters. Atto594 and Alexa594 were excited with a 561 nm laser and recorded with a 580–630 nm filter. iFluor647 was excited with a 640 nm laser and detected with a 650–720 nm filter. The pinhole size was set to 60 µm. For STED microscopy, pulsed STED lasers (595 nm for Alexa488 and 775 nm for Alexa594 and iFluor647) were used for depletion. STED images were recorded employing a time-gated detection with 0.75 ns delay and 8 ns gate width. Depending on the experiment, pixel size was set to 20–50 nm. Z-Stacks were acquired by recording images every 400 nm starting from the basal membrane of the cell and from then on moving up to 1600 nm above the basal membrane.

### Image analysis

For image analysis the program ImageJ was used. For Fig. 2d, rectangular ROIs fitted to the size of the aggregates were placed onto CD63/actin positive aggregates and the Pearson correlation coefficient (PCC) was calculated using a custom ImageJ macro. For Fig. 4, freehand ROIs were drawn outlining actin/CD63/CD151 positive aggregates. The average intensity within the ROIs was calculated and corrected for background. The same ROIs were used for calculating the PCC between CD63/actin, CD63/CD151, and CD151/actin. For Fig. 5, the PCC was calculated using squared ROIs placed on non-aggregated or aggregated actin structures (for all analyzed structures see Fig. S3).

### Electron microscopy analysis

The EM micrographs shown in Fig. 6 originate from the same preparation as previously published [15]. Here, we focus on endocytic vesicles which were not investigated in the previous study. In brief, HeLa cells were grown on a 50 µm thick, gas-permeable lumoxTM film (greiner bio-one), incubated with approximately 500 HPV16 PsVs per cell for 4 h, fixed and stained. Ultrathin sections were analyzed with a Zeiss EM 902 electron microscope, equipped with a TRS digital camera [15].

## Results

CD63, also known as Lysosome Associated Membrane Protein 3 (LAMP-3), is one of the few tetraspanins which is localized only to a low percentage at the plasma membrane. It is enriched in multivesicular bodies and vesicles trafficking between the cell membrane and lysosomes/late endosomes [34]. In HeLa cells, HPV16 PsVs associate with aggregated CD63 on the cell surface and localize to CD63 positive intracellular vesicles [15]. Moreover, in isolated basal membranes from keratinocytes, CD63 nanocluster-networks form at PsV attachment sites [16].

**Fig. 1:**
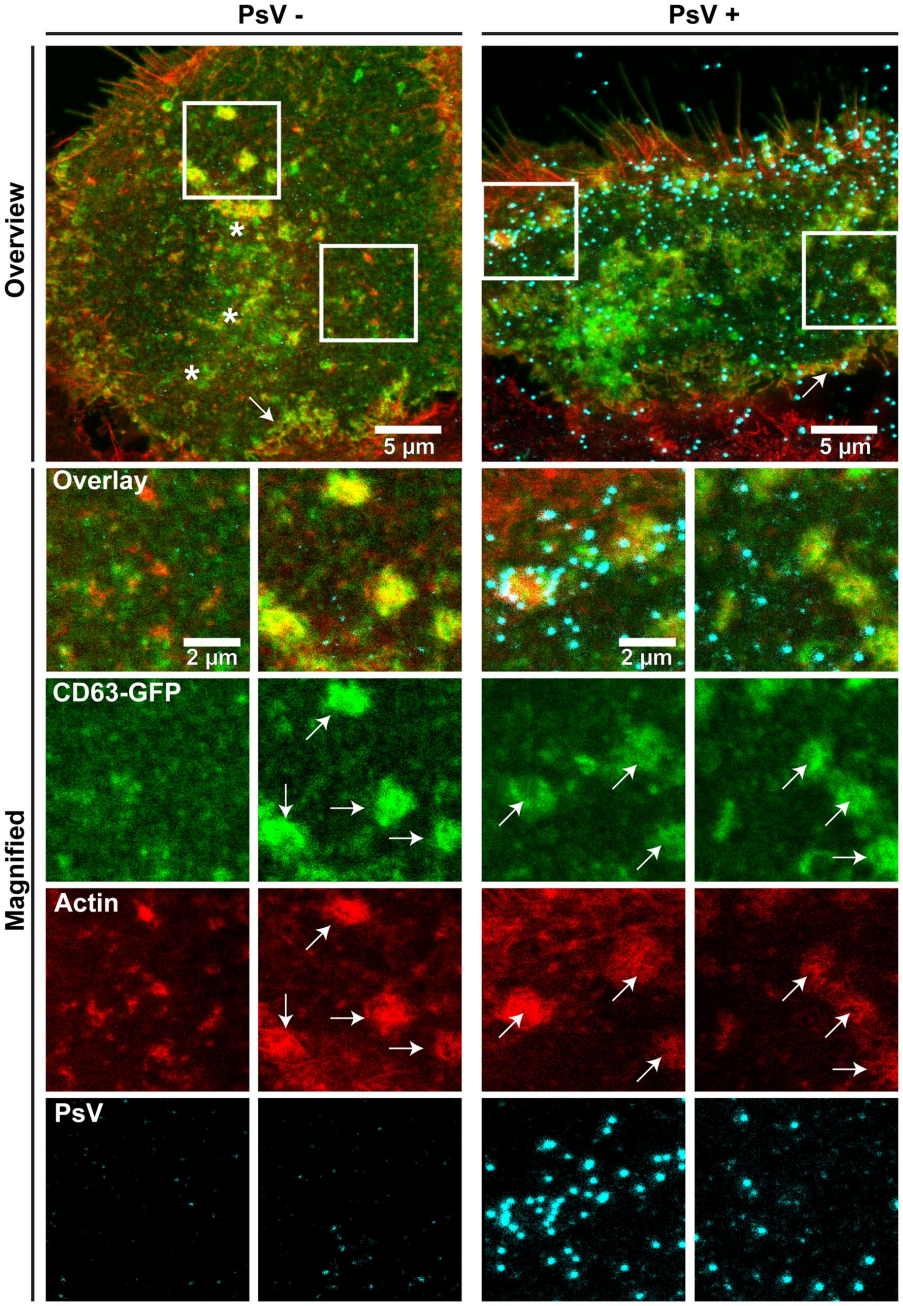
CD63/actin positive aggregates in HaCaT cells in the absence and presence of PsVs. HaCaT cells were transfected with CD63-GFP and 1 day later incubated for 3 h at 37 °C without (left panels) or with (right panels) PsVs. Cells were fixed and stained for PsVs and actin with an antibody and fluorescently labeled phalloidin, respectively, and GFP signal was enhanced by nanobodies. Confocal scans from the basal cell membrane were recorded. The linear lookup tables illustrate the channels for CD63-GFP, actin and PsVs in green, red and cyan, respectively. Overlap between green and red is illustrated in yellow and overlap between all three channels in white. Examples of patched structures counted as aggregates in Fig. 2 are marked by arrows in the magnified views. The most left magnified view illustrates patchy CD63-GFP structures that were not counted as aggregates. The two magnified views to the right show aggregates associated with many (left) and few (right) PsVs. For Fig. 2, aggregates were counted per cell base. Large continuous structures, like those often found at the cell periphery (see arrows in overview images) or CD63-vesicular structures in the perinuclear region (see asterisks) were excluded from the analysis

**Fig. 2:**
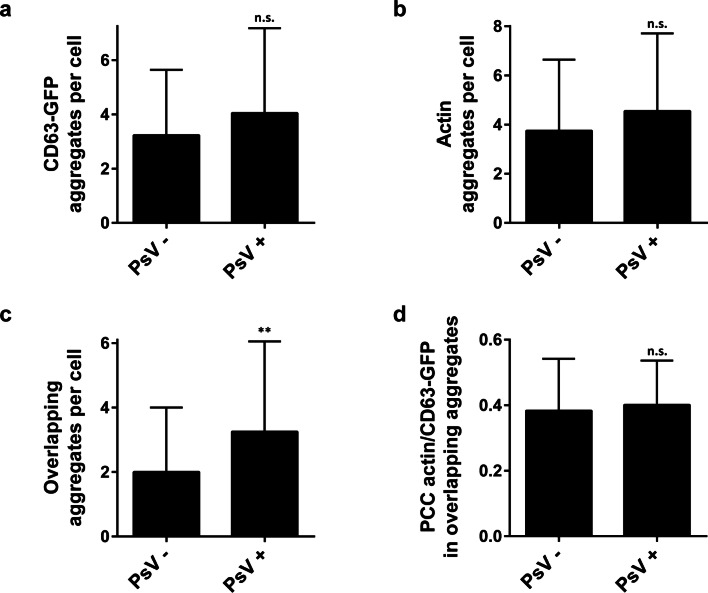
Quantification of CD63/actin positive aggregates in PsV treated and untreated HaCaT cells. The number of CD63 aggregates (**a**), actin aggregates (**b**), and of aggregates with overlapping CD63 and actin signals (**c**), which are a subset of (**a**) and (**b**), was counted manually in cells as shown in Fig. 1. **d** Regions of interest (ROIs) were placed on CD63/actin aggregates from PsV treated and untreated cells and the Pearson correlation coefficient (PCC) within the ROIs was calculated. Values are given as means ± SD (For **a**–**c**: *n* = 59–60 basal membranes collected from 3 biological replicates; for d: *n* = 120–195 overlapping aggregates as counted from **c**). **p < 0.01; *n.s.* not significant (unpaired Student’s *t*-test, comparing PsV treated to untreated cells)

We asked whether PsVs promote CD63 aggregates in the basal area of HaCaT cells that could be cell surface aggregates or vesicular structures close to the plasma membrane. HaCaT cells expressing CD63-GFP were incubated with PsVs for 3 h, washed, fixed, stained for actin and PsVs, and optical sections from the basal cell membrane were taken by confocal microscopy. A CD63-GFP fluorescent patch was counted as an aggregate if it was brighter and several times larger than other CD63 entities in its surroundings. Excluded from the analysis were large elongated structures often occurring at the cell periphery (Fig. 1; see arrows in overviews), accumulated CD63 at cell–cell contact sites, or CD63 vesicular structures often found around the nucleus (Fig. 1; see asterisks in left overview). We counted actin aggregates as well, employing the same criteria. As quantified in Fig. 2, CD63 aggregates are already present in the absence of PsVs, which is expected, as CD63 localizes to several types of intracellular organelles [34] that may be present in the subplasmalemmal area. An alternative explanation is that CD63 overexpression may promote tetraspanin patching (see also images in Fig. S1 comparing overexpressing and non-overexpressing cells). The number of patched structures was highly variable between individual cells (see also examples in Fig. S1). PsVs are not randomly scattered across the cell membrane but often concentrate at the cell periphery (see right overview in Fig. 1). Sometimes, several PsVs crowd together at locations at which CD63 and actin are co-patched (Fig. 1; see from condition PsV+ the left aggregate in the left magnified view). As CD63 is required for HPV intracellular trafficking [18] and actin polymerization appears to be required for vesicle scission [23], these structures could represent endocytic organelles. PsVs hardly increase the number of CD63 and actin spots (Fig. 2a, b), but those spots positive for both CD63 and actin increase by about 50% (Fig. 2c). On the other hand, in this subset of spots PsVs do not increase the signal overlap (Fig. 2d). These observations suggest that PsVs may promote the formation of endocytic organelles enriched both in CD63 and actin. However, these architectures per se are not special or novel types of organelles, but exist in the absence of PsVs as well.

**Fig. 3:**
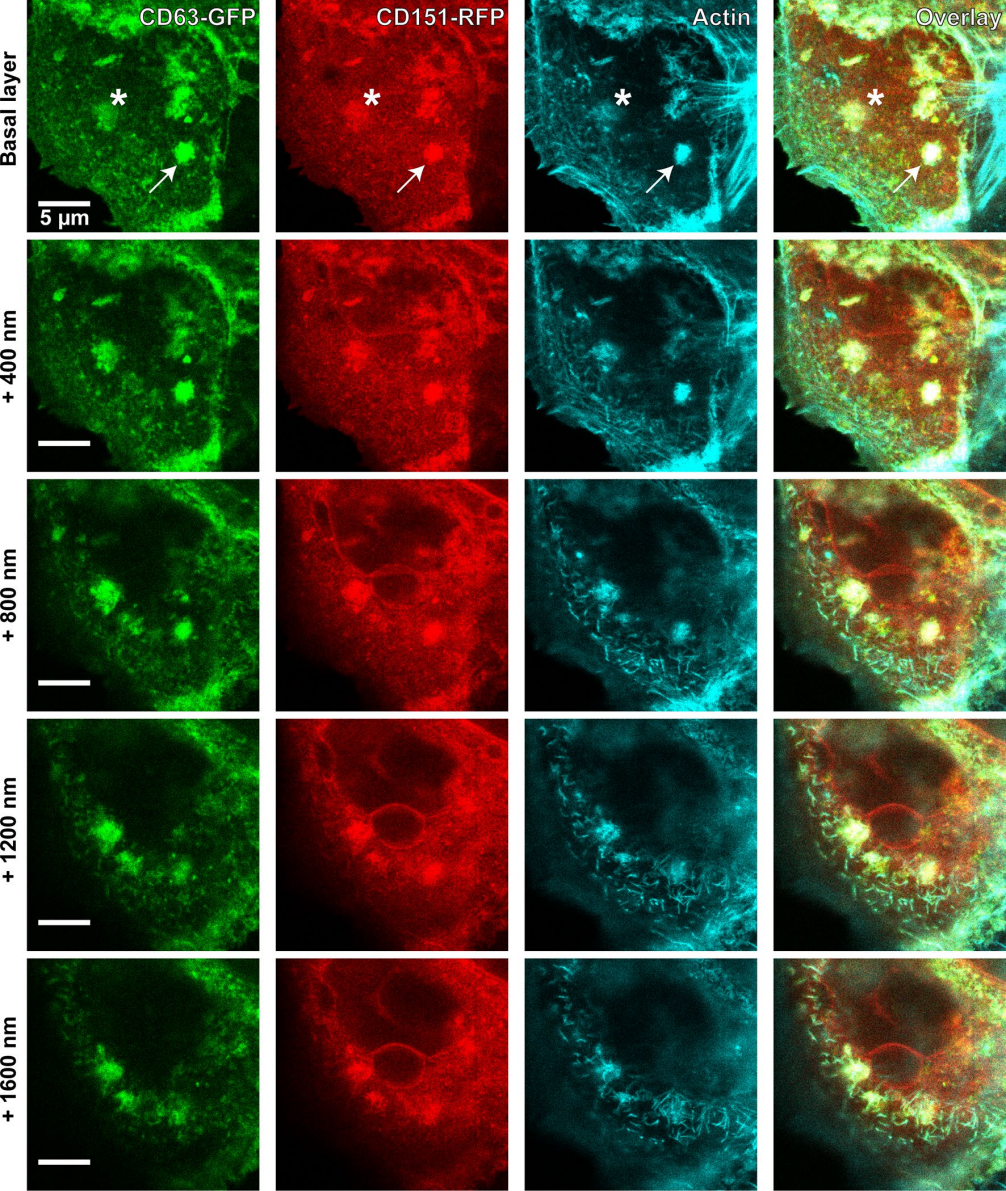
Aggregates enriched in CD63, CD151 and actin. HaCaT cells were transfected with CD63-GFP and CD151-RFP, and 1 day later incubated for 3 h at 37 °C with PsVs. Cells were fixed and stained for actin by fluorescently labelled phalloidin, and GFP- and RFP-signal was enhanced by nanobodies. An image stack was recorded starting at the basal cell membrane, with 400 nm axial distances between the optical sections. The linear lookup tables illustrate the channels for CD63-GFP, CD151-RFP and actin in green, red and cyan, respectively. Overlap between all three channels is illustrated in white. See Fig. 4 for the analysis of the relationship between the signals

**Fig. 4:**
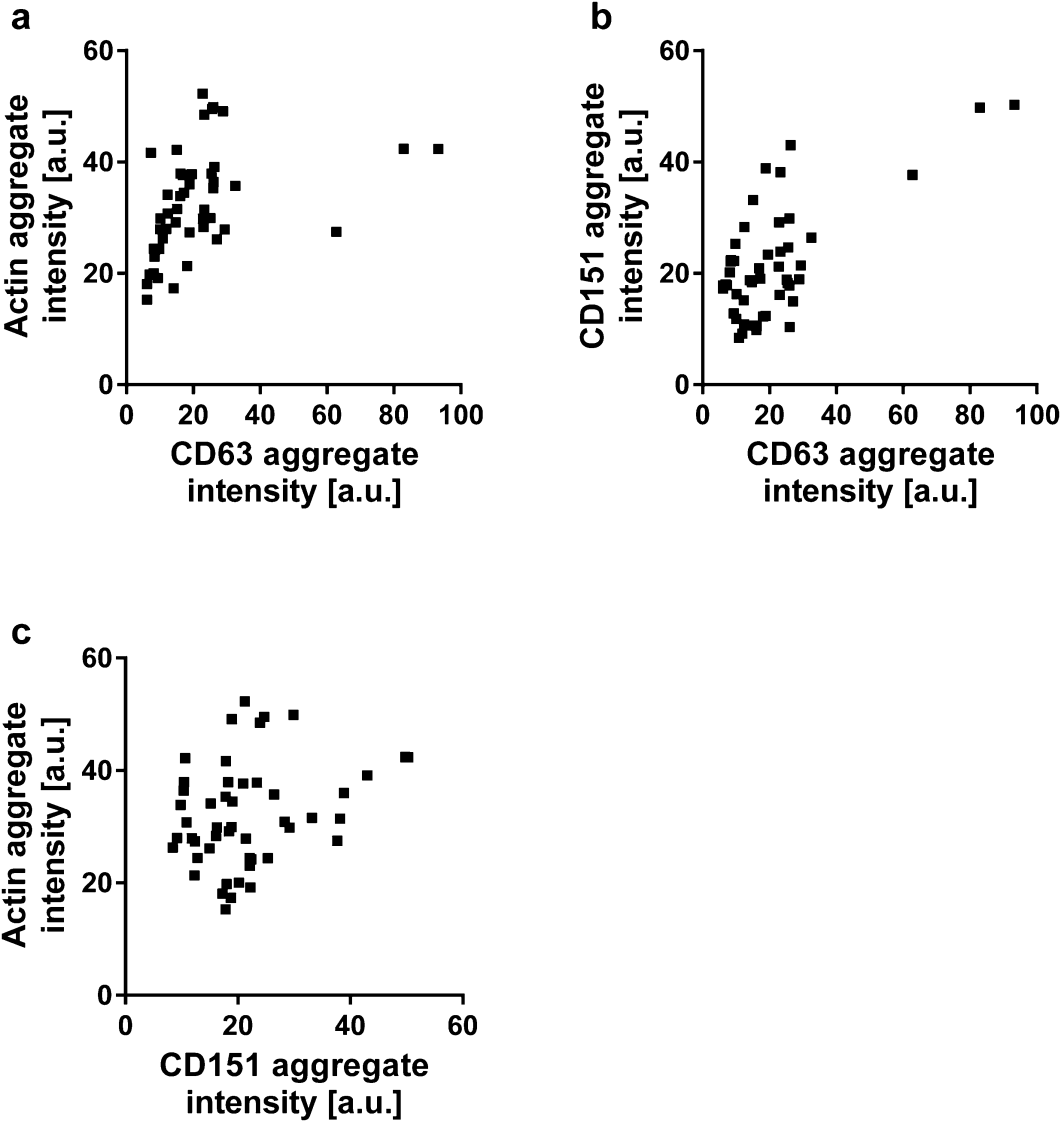
Relationship between the intensities of CD63, CD151 and actin aggregates. The intensities of actin and CD63 (**a**), CD151 and CD63 (**b**) and actin and CD151 (**c**) aggregates recorded from the basal layer, as shown in Fig. 3, were plotted against each other. From the same regions of interest the Pearson correlation coefficients (PCCs) were determined obtaining 0.44 ± 0.14 (CD63/actin), 0.39 ± 0.15 (CD63/CD151), and 0.41 ± 0.14 (CD151/actin) (values are given as means ± SD). (**a**–**c**: *n* = 49 aggregates from 20 cells collected from 3 biological replicates)

We next tested whether the CD63/actin-rich structures also contain the tetraspanin CD151. CD151 is a likely candidate, because it is essential for HPV entry in different cell types [3, 15, 19] and colocalizes with CD63 on the cell surface of HeLa cells [15]. HaCaT cells were co-transfected with CD151-RFP and CD63-GFP, incubated for 3 h at 37 °C with PsVs, fixed, stained, and imaged by confocal microscopy. Starting at the basal cell membrane, a stack of five optical sections was recorded, separated by an axial distance of 400 nm. Figure 3 shows an example illustrating all five scans. The uppermost section shows the basal cell membrane. Several tetraspanin aggregates are visible, which are spherical in shape and have diameters of a few micrometers in the lateral plane. In general, the intensities in the three channels displaying CD63, CD151 and actin correlate (Fig. 4), suggesting they mutually depend on each other. High overlap between signal pairs, as suggested by Pearson correlation coefficients ranging from 0.39 to 0.44, support the idea that in these aggregates all three components are enriched.

One aggregate displays a strong signal intensity in the plane of the cell membrane (marked with an arrow). Also in the sequential optical sections, the signal is strong up to 800 nm towards the interior of the cell, before it becomes fainter. Another aggregate in the green channel is less bright in the basal plane (marked with an asterisk). However, it increases in intensity at 400 and 800 nm, and the intensity remains high until 1600 nm. The weaker signal at the cell membrane may indicate that this is an endocytic organelle that has already pinched off from the cell membrane, representing a later stage in the pathway of endocytosis, whereas the first one may be still connected to the plasma membrane. These observations suggest that PsVs are internalized by large membrane invaginations strongly enriched with at least two-types of tetraspanins. These invaginations are coated intracellularly with polymerized actin and their lateral dimension seems to be not much different from their axial extension. However, the data do not allow a clear differentiation between spherical and tubular shapes.

There is no report on a direct interaction between tetraspanins and filamentous actin. Yet, the staining of filamentous actin matches well the tetraspanin signal (Fig. 3). This suggests a molecular link between the aggregated tetraspanins and intracellular actin, similarly to ezrin-radixin-moesin (ERM) proteins that connect CD81 and tetraspanin interaction partners to the actin cytoskeleton [35]. OBSL1, which is a cytoskeletal adaptor protein related to obscurin [26], is a candidate for such a molecular link because of several reasons. First, it interacts with the HPV16 capsid protein L2, second, it colocalizes with CD151, and third, it is required for HPV16 endocytosis [27].

**Fig. 5:**
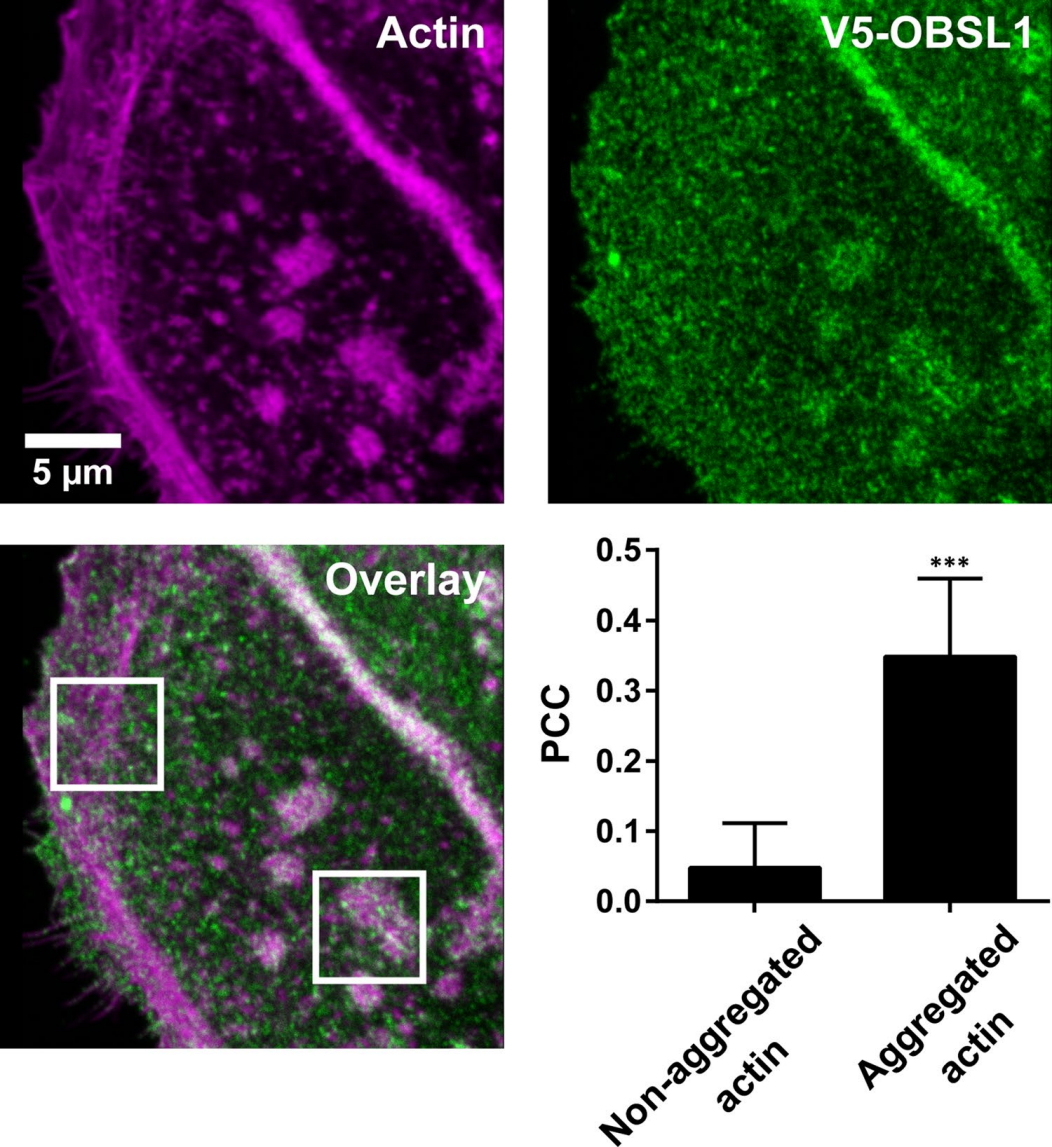
Overlap between actin and OBSL1. HaCaT cells were transfected with V5-OBSL1 and 1 day post transfection cells were incubated with PsVs for 3 h, fixed and stained for V5 with an antibody and for actin with phalloidin conjugated to a fluorophore. The linear lookup tables illustrate the channels for V5-OBSL1 and actin in green and magenta, respectively. The similarity between the OBSL1 and actin signals was quantified by calculation of the Pearson Correlation Coefficient (PCC). Values are shown as means ± SD (*n* = 21–27 ROIs, shown in Fig. S3, collected from 39 cells pooled from 4 biological replicates). ***, *p* < 0.001 (unpaired Student’s *t*-test, comparing aggregated to non-aggregated actin)

We studied the spatial relationship between OBSL1 and actin by overexpression of a V5-tagged OBSL1 construct. HaCaT cells were treated with PsVs, washed, fixed, stained, and confocal sections of the basal layer were imaged. Overexpression of OBSL1 had no obvious influence on actin, as both overexpressing and non-overexpressing cells have a comparable actin pattern (Fig. S2). The images were analyzed for the overlap between OBSL1 with actin, differentiating between non-aggregated, more filamentous actin structures usually found at the cell periphery (Fig. 5; see upper left white box in overlay) and aggregated signals (Fig. 5; see lower right white box in overlay), presumably organelle-associated F-actin. Only structures that could be clearly assigned to one of these classes were analyzed (for analyzed images see Fig. S3). Cell–cell-contact sites, where actin strongly accumulates alongside with OBSL1, were excluded from the analyses. As shown in Fig. 5, we found a striking difference in overlap between the two types of actin pools, suggesting that OBSL1 is preferentially associated with organelles.

**Fig. 6:**
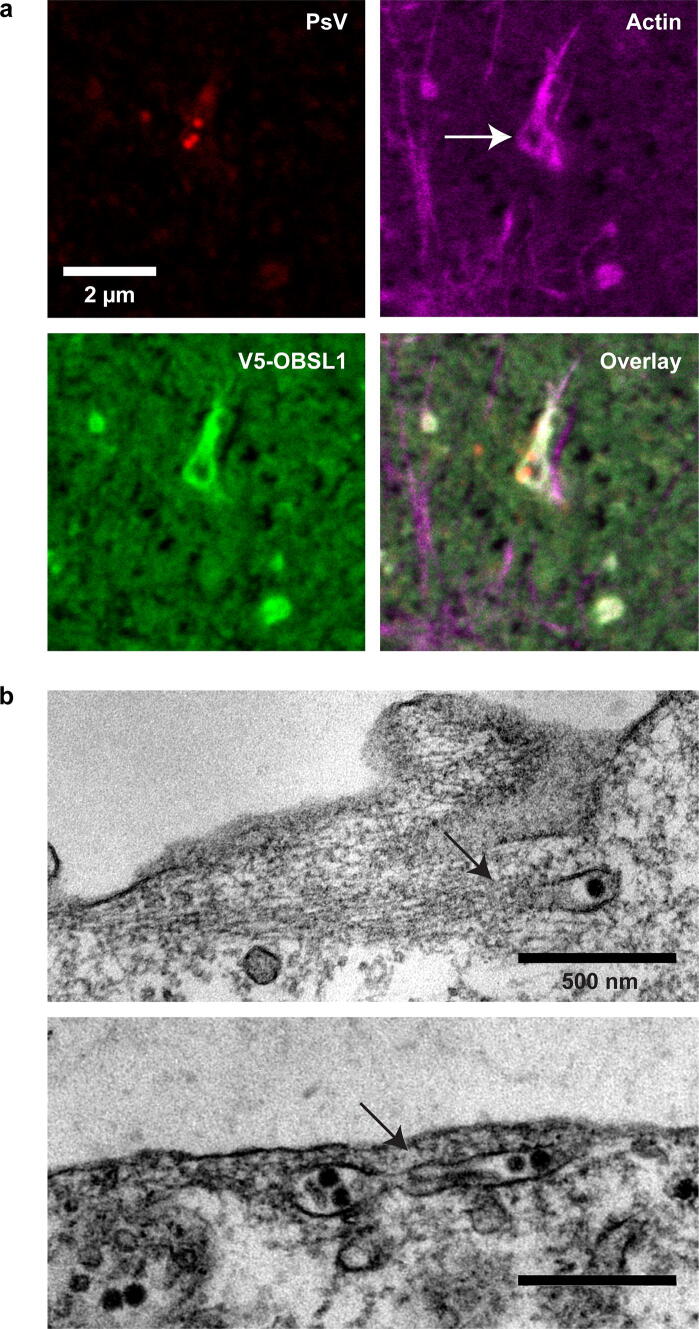
Morphology of endocytic organelles. **a** HepG2 cells were transfected with V5-OBSL1. One day post transfection, cells were incubated with PsVs for 3 h and then exposed to a brief ultrasound pulse, which removes the upper parts of the cells leaving behind the basal cell membranes. These membrane sheets were fixed and stained for V5 and PsVs with antibodies, and for actin with phalloidin conjugated to a fluorophore. The linear lookup tables illustrate the channels for PsV, V5-OBSL1 and actin in red, green and magenta, respectively. Images were screened for locations at which PsVs are close to actin and OBSL1 positive structures. Shown is the largest example we found. **b** Electron micrographs of HeLa cells incubated with HPV16 PsVs (visible as black dots with a size of 50–60 nm). The lower image shows a roughly 800 nm long tubular and virus filled endocytic vesicle. Filamentous actin (see arrows) is in close proximity to the virus containing organelles. Images are taken from an experiment previously described [15]

Next, we were interested whether PsVs would be present at actin-rich sites forming in the moment of membrane invagination, which could be defined by OBSL1. To be sure that we study early stages at the cell surface, prior to fission from the cell membrane, we examined membrane sheets. Membrane sheets were generated from cells expressing the OBSL1 construct, which were incubated for 3 h with PsVs prior to membrane sheet formation. Because the OBSL1 construct did not express well in HaCaT cells, and because HaCaT cells are quite resistant to membrane sheet generation, we used HepG2 cells for this experiment. The upper cellular parts were removed by a short ultrasound-pulse, leaving behind only the basal cell membrane that remains adhered to the glass-coverslip. Membrane sheets were fixed, stained and imaged with superresolution STED microscopy. The recorded images were screened for locations with PsVs close to actin and OBSL1 positive structures. Figure 6 shows the largest plasmalemmal architecture found in this initial screening experiment. In this one example, actin forms an irregular, elongated structure (marked by an arrow in Fig. 6a). Moreover, the microscope resolves a central hole, indicating the membrane has not yet pinched off. This likely is an early stage of the endocytic event, at which the viral platform invaginated into the cell interior to some extent. In this case, the removal of the upper cellular part would have ripped off the invaginating organelle and left behind the central hole with the proteins present in the moment of membrane invagination. Strikingly, the filamentous actin perfectly overlaps with OBSL1, and the OBSL1 ring-like structure is closely attached to the PsVs. Yet, there are two more, smaller actin structures (see upper left and lower right quarter in Fig. 6a), overlapping with OBSL1, but not with PsVs. Our experimental conditions did not allow finding more of such large architectures, pointing towards the possibility that large aggregates are much more frequent under overexpression conditions. Alternatively, in the moment of membrane sheet generation most of the endocytic structures have pinched off already. However, the example suggests extensive overlap between OBSL1 and actin already at the cell membrane. In any case, in the future this anecdotal observation should be reproduced in a cell line more relevant for the study of HPV infection.

To get an idea about the size of early endocytic compartments we also examined electron micrographs from HeLa cells that did not overexpress tetraspanins. As suggested in a previous study, the compartments containing PsVs tend are in a range of 100–200 nm in diameter [23], much smaller than those under our overexpression conditions (Fig. 1 and Fig. 3). Still, we detected an up to 800 nm long tubular and virus filled structure (Fig. 6b, lower panel). Moreover, filamentous actin was found in close proximity to endosomes containing virus particles (Fig. 6b). Hence, the large endosome in close proximity to filamentous actin could represent the structures identified by fluorescence microscopy.

## Discussion

### Are HPVs engulfed by a tetraspanin web?

The evidence that tetraspanins form large networks of interactions initially came from biochemical experiments, showing that certain complexes are resistant to detergents [36, 37]. In recent years, microscopic studies have not only confirmed the existence of tetraspanin networks, but also revealed a novel layer of organisation. It appears that tetraspanins segregate into ≈ 100 nm large nano-clusters that group together to form larger cluster assemblies. The composition and size of these assemblies needs to be characterised in more detail.

How many of the PsV particles associate with CD63 aggregates? From images as shown in Fig. 1, we estimate that less than 10 % of the PsVs overlap with CD63 aggregates. However, this is a strong underestimate because it excludes very large accumulations, where single patches are hardly discernible and which are often found at the cell periphery where the density of PsVs is high, cell–cell contact sites or vesicle belts around the nucleus.

It is possible that TEMs, as defined by biochemical approaches, are tetraspanin cluster networks that also incorporate tetraspanin interaction partners. The size of these networks—or the size of the tetraspanin web—could depend on the availability of cellular components (e.g., be influenced by tetraspanin overexpression), extracellular binding partners like the virus particles (which due to their structural periodicity could have additional crosslinking effects), and the signalling state of the cell (which could modulate the tetraspanin web via intracellular dynamics). Here, we did not study all these issues. We only find that at least two different tetraspanins co-aggregate and by this are capable of covering a large area of several square micrometres. This specialized area apparently forms an invagination, can pinch off from the cell membrane and becomes an intracellular organelle, a process likely driven by actin dynamics.

Altogether, the data is consistent with the idea that PsVs associate with a tetraspanin web. In this web the tetraspanins would organize a platform [10] with different components such as OBSL1 for linking the virus to the actin cytoskeleton, or a receptor for virus attachment, e.g., integrin α6 that promotes papillomavirus binding [10, 38, 39]. It remains unclear whether the PsVs induce a special tetraspanin web or whether they associate to cluster crowds that are already present prior to virus contact.

Tetraspanin crowds have also been documented for CD9 during the infection of coronaviruses and influenza viruses, where they colocalize with the respective receptor molecules and pseudoviral particles [5]. Here, we identify CD151, CD63 and actin as components of tetraspanin crowds associated with HPV16 PsVs. Although not shown directly, the data further suggest that OBSL1 is part of the platform, which is in line with its requirement for HPV endocytosis and association with CD151 [27]. We also observed that OBSL1 overlaps with actin at cell–cell contact sites (Fig. 5). Interestingly, CD151 was previously shown to accumulate at cell–cell contact sites strongly colocalizing with actin [40]. This could indicate that OBSL1 has a strong preference for sites, where CD151 regulates cytoskeletal reorganization.

Our results rely on overexpression. For instance, CD63 is predominantly located intracellularly and we needed to increase the amount in the plasma membrane for proper visualization of plasmalemmal aggregates. Moreover, a previously employed antibody against OBSL1 [27] was not commercially available, requiring OBSL1 overexpression. Therefore, at some point the results should be verified under non-overexpression conditions. Due to their association with the tetraspanin web, more components such as ERM proteins [35] and GTPases [41] could be part of the platform. However, this needs to be shown in the future and additionally the concept needs to be verified in more cell lines.

### Size of the endocytic compartments

Intracellular trafficking vesicles have a quiet variable size range up to a micrometer [18, 23]. Using electron microscopy, previous reports have shown that in HeLa and HaCaT cells endocytic compartments with PsVs are rather small, in the 100 nm range [23]. Only upon treatment with cytochalasin D, inhibiting actin polymerization, they grow larger, into tubes in length of up to microns, still connected to the cell membrane [23].

In this study, it appears that early vesicles, still located close to the cell membrane have a size in the range of a few micrometers in diameter (Figs. 1 and 3), which is larger than observed in electron microscopy [23]. At this point, it is not clear what accounts for the difference. Likely, overexpression of tetraspanins leads to the formation of excessively large endocytic compartments that would not form at endogenous tetraspanin levels (Fig. S1). This suggests that the efficiency of tetraspanin web building may control the size of the forming endocytic organelles. However, despite of such a possible overexpression artefact, the basic mechanism—tetraspanins organizing a viral entry platform—is plausible. Interestingly, antibody induced co-aggregation of tetraspanins and actin has been reported in epidermal carcinoma A431 cells [40], which resemble the aggregates we can observe in our experiments. Moreover, it was shown that, depending on the cell maturation status, TSPAN7 regulates actin nucleation in the context of HIV-1 transfer from dendritic cells to T-cells [42, 43]. This supports the idea that tetraspanin webs are able to induce actin reorganization which is a prerequisite for viral entry [23–25], possibly via OBSL1.

## Conclusion

Tetraspanins are master organizers of the cell membrane. The architectures they build are required for cell entry of virus particles and contain a mixture of tetraspanins and can be considered as viral entry platforms.

At this point it is not clear whether viral particles switch on a mechanism that causes massive aggregation of tetraspanin molecules into a large web, or whether they bind to architectures already present prior to the contact of the virus with the cell membrane.

## Electronic supplementary material

Below is the link to the electronic supplementary material.

Supplementary file1 (PDF 681 kb)
