# Supplementary material for: HPV caught in the tetraspanin web?
Source: Med Microbiol Immunol. 2020 Jun 13;209(4):447–59. doi: 10.1007/s00430-020-00683-1 (PMC7293171; doi:10.1007/s00430-020-00683-1)
Supplement: Supplementary file 1 — Supplementary file1 (PDF 681 kb) [file 430_2020_683_MOESM1_ESM.pdf]

Electronic supplementary material

HPV caught in the tetraspanin web?

Jérôme Finke[1], Lisa Hitschler[1], Klaus Boller[2], Luise Florin[3] and Thorsten Lang[1]

[1]Department of Membrane Biochemistry, Life & Medical Sciences (LIMES) Institute, University of Bonn, Carl-Troll-Straße 31, 53115 Bonn, Germany, [2]Paul Ehrlich Institute, Paul-Ehrlich-Straße 51-59, 63225 Langen, Germany, [3]Institute for Virology and Research Center for Immunotherapy (FZI), University Medical Centre of the Johannes Gutenberg University Mainz, Obere Zahlbacher Straße 67, 55131 Mainz, Germany

[*]Correspondence should be addressed to Thorsten Lang (thorsten.lang@uni-bonn.de) or Jérôme Finke (s6jefink@uni-bonn.de)

This article is part of the Special Issue on Tetraspanins in Infection and Immunity.

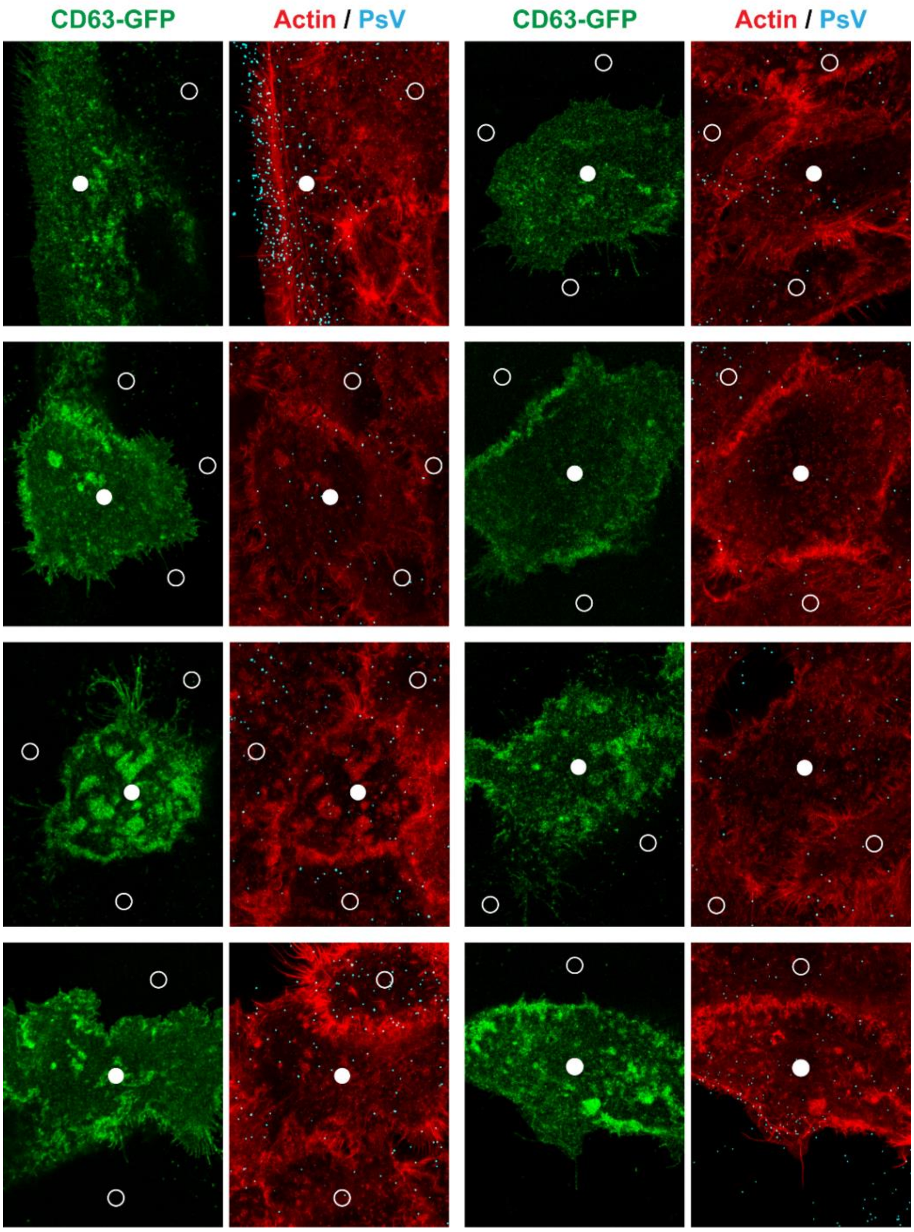

Supplemental Fig. 1 *Actin distribution in CD63 overexpressing and non-overexpressing cells.*

Set of images from the experiment shown in Fig. 1 and Fig. 2. The linear lookup table visualizes the channel intensities for CD63-GFP, actin and PsVs in green, red and cyan, respectively. PsV and actin channels are displayed at identical intensity scaling for all images. Due to great variability in the expression level, CD63 images were scaled individually. In each image center, a cell overexpressing CD63 is present (marked with a filled circle) together with at least one neighboured cell not overexpressing CD63 (marked with an open circle). No striking difference was observed regarding phalloidin-stained F-actin level or distribution, with the exception of more actin aggregates in CD63 overexpressing cells that overlap with CD63. This points to the possibility that tetraspanin overexpression promotes tetraspanin patching (see discussion).

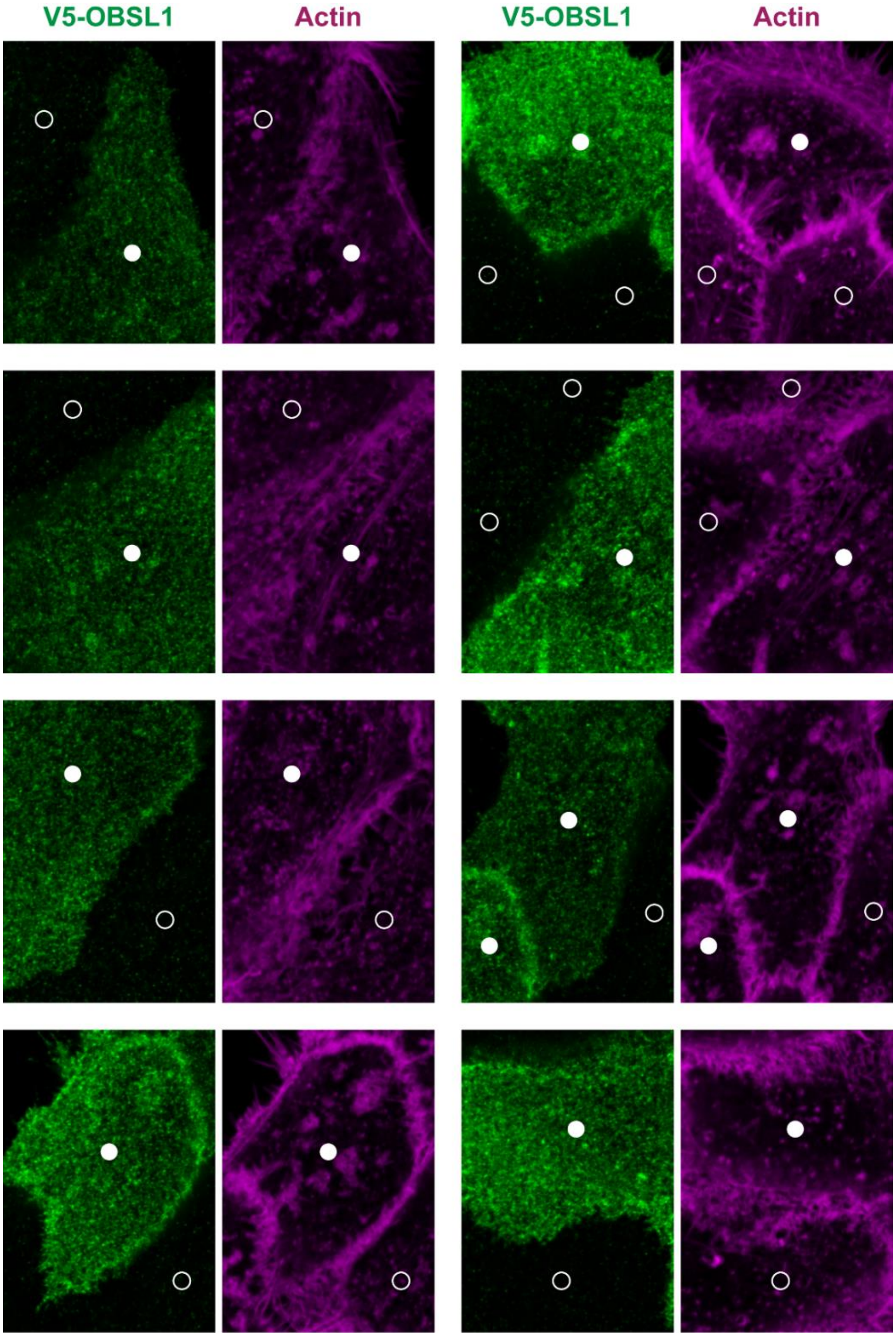

Supplemental Fig. 2 *OBSL1 overexpression has no influence on the overall actin distribution.*

Set of images from the experiment shown in Fig. 5. The linear lookup tables illustrate the channels for V5-OBSL1 and actin in green and magenta, respectively. The actin channel is displayed at identical intensity scaling for all images. Due to great variability in the expression level, OBSL1 images were scaled individually. Each displayed image shows a cell overexpressing OBSL1 (marked with a filled circle) with at least one adjacent cell not overexpressing OBSL1 (marked with an open circle). No striking difference was observed for actin distribution in cells overexpressing OBSL1.

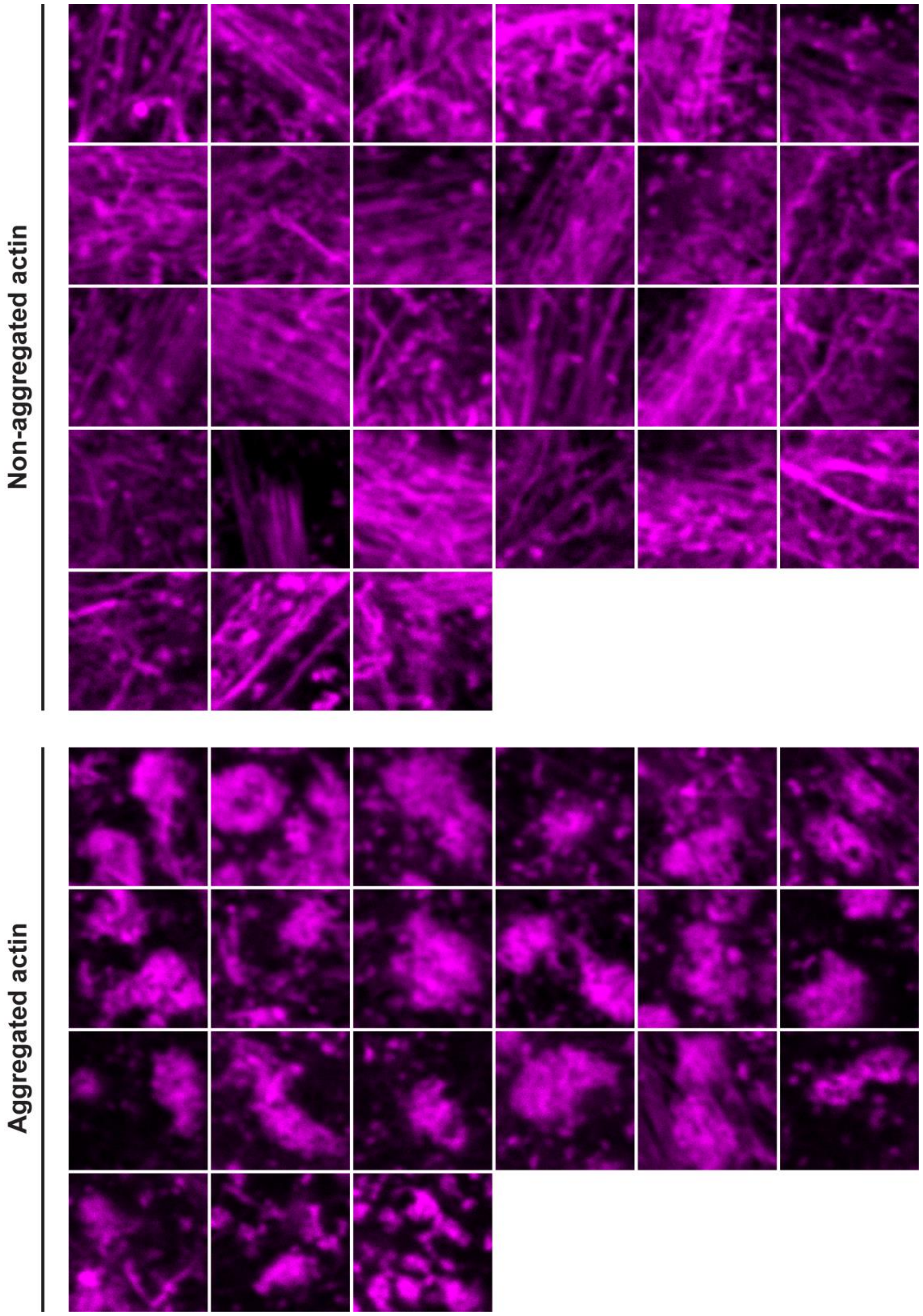

Supplemental Fig. 3 *Regions analyzed for Fig. 5.*

All regions of interest analyzed in Fig. 5 are displayed. Actin organizes in non-aggregated, filamentous structures (upper panels) or aggregates (lower panels).
